# Endo-MedSAM: a promptable vision foundation model adaptation for uterus segmentation on pelvic MRI in endometriosis

**DOI:** 10.3389/frph.2026.1790980

**Published:** 2026-05-25

**Authors:** Rawan AlSaad, Shima Albasha, Rajat Thomas

**Affiliations:** 1 Weill Cornell Medicine-Qatar, Doha, Qatar; 2 Division of Reproductive Medicine, Hamad Medical Corporation, Doha, Qatar

**Keywords:** artificial intelligence, endometriosis, MedSAM, MRI, segmentation, uterus, vision foundation model, women's health

## Abstract

**Background:**

Endometriosis is a common gynecologic condition in which pelvic MRI plays an important role in diagnosis and preoperative assessment. AI-enabled automated uterus segmentation on pelvic MRI could support endometriosis care by enabling standardized volumetric measurements and quantitative imaging analyses. However, developing robust AI models for this task is challenging because endometriosis often distorts pelvic anatomy through adhesions, uterine displacement, and coexisting fibroids or adenomyosis. Although promptable medical vision foundation models, such as MedSAM-2, provide a promising framework for interactive segmentation, their zero-shot performance on endometriosis MRI remains limited.

**Objective:**

To develop Endo-MedSAM, a uterus-focused adaptation of MedSAM-2 for pelvic MRI in endometriosis, and to systematically evaluate its segmentation performance across institutions and prompting strategies.

**Methods:**

We used a pelvic MRI dataset comprising 74 subjects and 3,449 T2-weighted slices from two institutions (D1, multicenter; D2, single-center). Endo-MedSAM was initialized with MedSAM-2 weights and fine-tuned by training the prompt encoder and mask decoder while unfreezing the final layers of the image encoder. Slice-wise predictions were then reconstructed into 3D volumes for volumetric evaluation. Performance was assessed using slice-level and 3D Dice scores and the 95th percentile Hausdorff distance (HD95).

**Results:**

Endo-MedSAM was evaluated in three configurations: training on the single-center D2 cohort and testing on the multicenter D1 cohort, training on D1 and testing on D2, and using a mixed D1 + D2 split with 75% of patients for training and 25% for testing. Across these settings, Endo-MedSAM achieved mean 3D Dice scores of 0.81–0.88 with bounding-box prompts and 0.68–0.76 with 1-click and 2-click point prompts. Compared with zero-shot MedSAM-2, this represented absolute 3D Dice improvements of approximately 0.27–0.34 for bounding-box prompting. Endo-MedSAM also showed markedly better performance across all prompting modes, with the largest gains observed for bounding-box prompting.

**Conclusion:**

Endo-MedSAM achieved robust uterus segmentation on endometriosis pelvic MRI, consistently outperforming zero-shot MedSAM-2 across both multicenter and single-center settings while supporting bounding-box and low-interaction point prompting. Clinically, this can enable faster and more reproducible uterus delineation, reduce manual contouring burden, standardize measurements across scanners and sites, and support downstream quantitative imaging workflows in endometriosis care.

## 1 Introduction

Endometriosis is a chronic, estrogen-dependent inflammatory disease in which endometrium-like tissue grows outside the uterine cavity, affecting an estimated 10% (≈190 million) of women of reproductive age worldwide (1). It is a leading cause of chronic pelvic pain, dysmenorrhea and subfertility, with significant impacts on quality of life, mental health and work productivity (2). Diagnosis typically relies on a combination of clinical history, pelvic examination, and pelvic imaging, most commonly transvaginal ultrasound and pelvic MRI, with laparoscopy used when indicated for definitive assessment and treatment planning (3). However, heterogeneous symptoms and limited specificity of imaging findings contribute to diagnostic delays of 4 to 12 years in many health systems, perpetuating under-diagnosis and delayed treatment (1, 4).

In pelvic MRI, uterus segmentation for endometriosis is clinically important yet anatomically challenging (5). Accurate delineation of the uterus provides a key anatomic reference for localizing disease, standardizing pelvic measurements, and enabling quantitative MRI analyses. However, manual segmentation is time-consuming and subject to inter- and intra-rater variability, particularly when anatomy is distorted and imaging protocols vary across sites and scanners. Automated uterus segmentation offers a practical alternative by providing fast, reproducible contours that reduce manual workload, improve measurement consistency, and support downstream workflows such as volumetric assessment, longitudinal monitoring, quantitative imaging analyses, and preoperative planning in endometriosis care.

In parallel, deep learning has been increasingly explored for pelvic MRI analysis and endometriosis characterization (6). On pelvic MRI, most prior work has focused on pelvic organ segmentation in broader gynecologic populations rather than endometriosis specific cohorts, including uterus and fibroid segmentation, pelvic organ delineation, and multi organ reproductive tract analysis (7–10). In contrast, endometriosis focused deep learning studies have largely targeted lesion detection or classification in ultrasound and laparoscopic images, including models trained on ultrasound examinations and laparoscopic dataset (11–15). To date, no existing models specifically address uterus segmentation in women with endometriosis, where distorted pelvic anatomy, adhesions, variable uterine position and shape, coexisting fibroids or adenomyosis, and heterogeneous MRI protocols across scanners and centers make this task particularly challenging (11, 18, 22).

The introduction of the Segment Anything Model (SAM) (16) marked a major milestone in image segmentation. SAM generates object masks from simple prompts, such as points or bounding boxes, without task-specific training, enabling strong zero-shot performance. In medical imaging, early studies adapted SAM by fine-tuning selected components or designing medical-specific prompts to better accommodate anatomical structures and clinical imaging characteristics (17–22). Medical SAM 2 (MedSAM-2) (23) extends promptable segmentation by unifying image-based inference and video-style mask propagation formulations for medical data.

In this work, we introduce Endo-MedSAM, a uterus-focused adaptation of the MedSAM-2 vision foundation model for endometriosis pelvic MRI, using a slice-wise image-based formulation and assessing volumetric accuracy by reconstructing 3D masks from the per-slice predictions. Our objective is to develop and evaluate an automated pipeline that leverages promptable foundation models to generate robust, clinically useful uterus segmentations across heterogeneous, real-world datasets. Specifically, we fine-tune MedSAM-2 using 3,449 pelvic MRI slice-mask pairs from two complementary real-world cohorts of women with endometriosis: (i) a multicenter dataset spanning multiple vendors, field strengths, and imaging protocols, and (ii) a single-center dataset acquired on a single MRI scanner. We benchmark Endo-MedSAM against zero-shot SAM-2 and nnU-Net baselines, and evaluate performance at both the slice level and volume level per patient, with the goal of enabling downstream quantitative analysis and supporting surgical planning.

## 2 Methods

### 2.1 Dataset

Our study used a publicly available pelvic MRI dataset (24) comprising scans from two clinical institutions: the Memorial Hermann Hospital System and the Texas Children's Hospital Pavilion for Women, as described in Table 1. The first dataset included MRI scans and corresponding uterus labels from 35 patients imaged before 2022. These examinations were acquired in routine clinical practice across 15 different sites, using eight scanner models from three vendors (GE, Philips, Siemens) at two field strengths (1.5 T and 3 T). All patients in this dataset were referred for MRI with a clinical suspicion of endometriosis, although a small subset were ultimately not diagnosed with the condition. The second dataset comprised MRI scans and labels from 39 patients with confirmed endometriosis imaged in 2022 at a single center using a Philips Ingenia 1.5 T scanner. For both datasets, we restricted our analysis to T2-weighted pelvic MRI sequences, which are routinely used for endometriosis assessment and uterus delineation.

**Table 1:** Dataset overview: endometriosis pelvic MRI cohorts.

Dataset	Clinical Institution	Sites (n)	Distinct MR scanner models (n)	Subjects (n)	T2-weighted slices (*n*)	Max no. of raters per subject
D1	Memorial Hermann Hospital System	15	8^a^	35	1,583	3
D2	Texas Children's Hospital Pavilion for Women	1	1^b^	39	1,866	1
Total		16	8	74	3,449	3

a Philips Achieva, Philips Ingenia, Philips Ingenia Ambition S, GE SIGNA EXCITE, GE SIGNA HDe, GE SIGNA Voyager, Siemens Espree, Siemens Verio.

b Philips Ingenia.

Uterus segmentations were generated in 3D Slicer by trained raters following a standardized contouring guideline. For the first dataset (D1), three raters manually contoured the uterus, prioritizing T2-weighted sequences while using additional sequences as needed to clarify anatomy. An experienced abdominal radiologist developed the segmentation protocol and reviewed the final labels from all raters, making corrections where necessary. Among the 35 subjects, 12 (34.3%) were annotated by three raters, 15 (42.9%) by two raters, and 8 (22.9%) by one rater. For the second dataset (D2), the uterus was manually contoured by an obstetrician–gynecologist assistant under the supervision of an expert gynecologist, using a consistent T2-weighted fat-suppressed protocol. Inclusion criteria for both datasets were: (1) suspected or confirmed endometriosis, (2) availability of a T2-weighted pelvic MRI, and (3) manual segmentation of the uterus by at least one rater.

### 2.2 Data preprocessing

All preprocessing was applied to T2-weighted pelvic MRI volumes and corresponding uterus masks in NIfTI format. For both datasets, each volume and mask were cropped to a standardized 256 × 256 in-plane field of view centered on the pelvis to reduce scanner/protocol variation while preserving the original slice thickness and inter-slice spacing. In Dataset 1 (D1), up to three raters provided uterus masks per subject; we constructed a voxel-wise soft consensus by averaging the binary masks [values in (0,1)] and thresholded at ≥0.5 to obtain a single binary reference label for training and evaluation. Dataset 2 (D2) contained a single expert-supervised uterus mask per subject and required no consensus aggregation.

After cropping and label preparation, uterus-positive slices were identified from each 3D mask. Slices without uterus pixels (mask sum = 0) were excluded from training and validation, and evaluation was likewise focused on uterus-positive slices for slice-level metrics. Data were split at the patient level into training, validation, and test sets to ensure subject-level independence. Before model input, each 2D slice was intensity-normalized using a robust percentile scheme: values were clipped to the 1st–99th percentiles, linearly rescaled, and converted to 8-bit to reduce outlier effects and harmonize intensity ranges across scanners and protocols.

### 2.3 Model architecture

Endo-MedSAM is built on the MedSAM-2 framework and operates on 2D axial slices extracted from cropped 3D T2-weighted pelvic MRI volumes. For each slice, the input to the network consists of a cropped normalized pseudo-RGB image (three identical channels derived from the T2 slice) together with a uterus prompt (bounding box or point-based prompt) derived from the reference mask during training and evaluation to simulate user prompts; in clinical deployment, prompts would be user-defined.

A core component of Endo-MedSAM is the SAM-2 image encoder, which generates a dense embedding for each MRI slice. We initialize the encoder with MedSAM-2 weights and freeze most of its parameters to retain the general medical segmentation capabilities learned during pre-training. To allow modest uterus-specific adaptation while limiting overfitting on a relatively small endometriosis cohort, we unfreeze only the final ∼10% (“tail”) of the encoder. We then fine-tune the prompt encoder and mask decoder for task-specific learning under strong regularization.

Although MedSAM-2 supports volumetric auto-tracking via a video-style formulation, we deliberately adopt an image-based, 2D slice-wise training strategy. This design reflects (i) the limited number of available 3D endometriosis volumes, (ii) heterogeneous multicenter acquisition and inter-slice variability that can degrade propagation consistency, and (iii) increased overfitting risk when training higher-capacity volumetric adaptations on small datasets.

**Figure 1:**
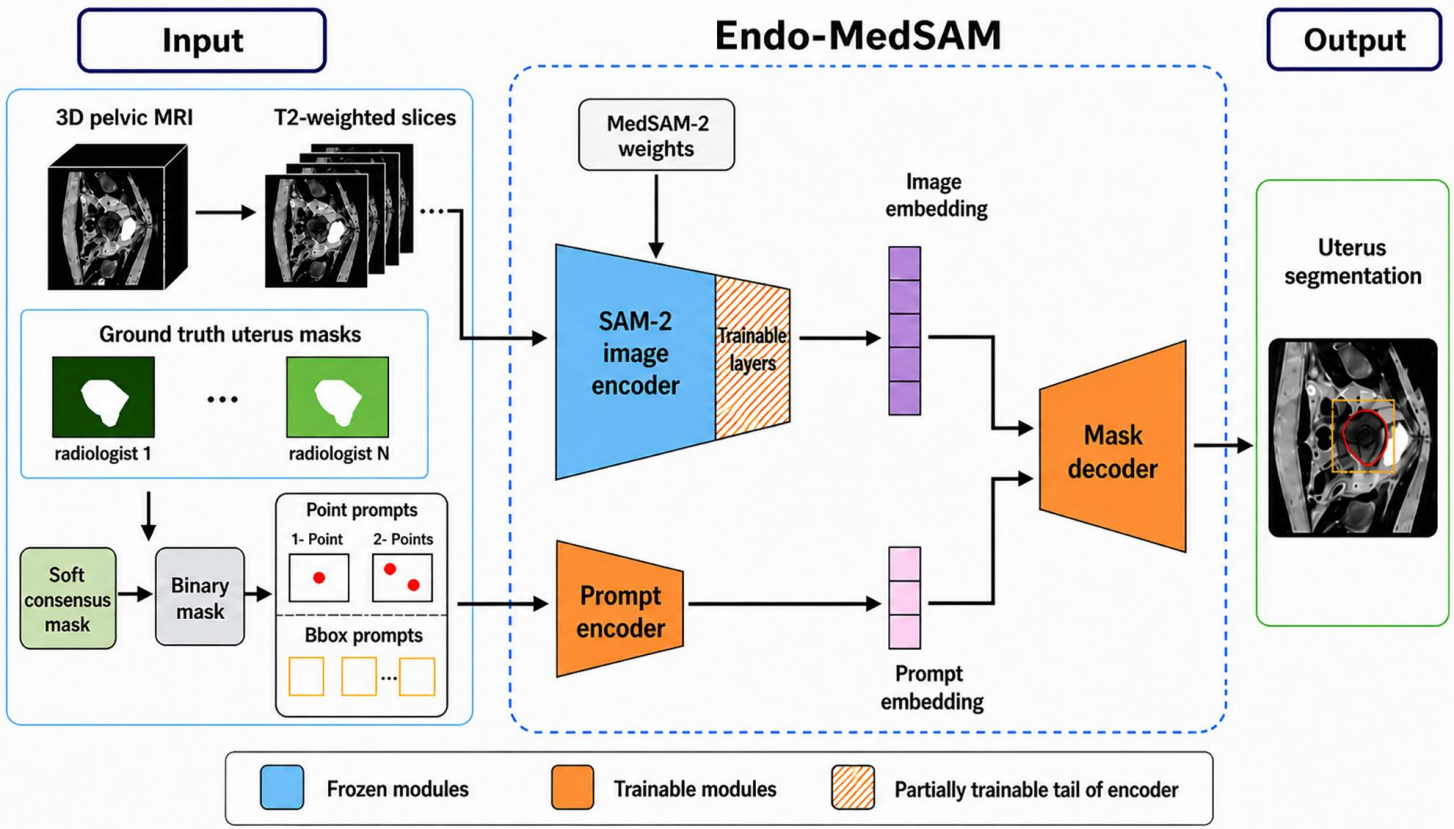
Endo-MedSAM framework for uterus segmentation from 3D pelvic MRI.

Prompt information is processed by the SAM-2 prompt encoder. Bounding boxes are converted into two corner points in image coordinates, and one-point or two-point prompts are similarly encoded as sparse spatial cues. The prompt encoder maps these inputs into low-dimensional prompt embeddings, which are then combined with the image embedding in the SAM mask decoder. The mask decoder produces a low-resolution logit mask that is upsampled back to the original 256 × 256 resolution to generate a 2D uterus segmentation for each slice. Slice-wise predictions are later reassembled into 3D volumes for evaluation, as illustrated in Figure 1. In our adaptation, all parameters of the prompt encoder and mask decoder are trainable, whereas the remaining SAM-2 modules are kept frozen except for the partially trainable tail of the image encoder.

### 2.4 Fine-Tuning and inference

Endo-MedSAM was fine-tuned in a 2D slice-wise mode on cropped T2-weighted images. For each training slice $x\in R^{H\times W}$ with binary uterus mask $y\in \{0,1{\}}^{H\times W}$, a tight 2D bounding box was computed from y and isotropically expanded by a random scale factor s∼U(1.2,1.7) for cropping; during validation, the expansion factor was fixed to s=1.5. Depending on the prompting mode, either the bounding-box corners or sampled point prompt(s) were encoded and passed to the SAM-2 prompt encoder, while the normalized slice served as input to the SAM-2 image encoder. Only uterus-positive slices were used for optimization; predictions were generated slice-wise and reassembled into subject-level 3D masks for volumetric evaluation using uterus-positive slices only. Slice-level Dice was computed on uterus-positive slices (i.e., slices where the reference mask contains uterus voxels), consistent with the slice set used for optimization. Volumetric (3D) Dice was computed per subject after stacking uterus-positive slice predictions into a 3D mask in the original slice order and comparing it with the reference 3D mask.

The model was initialized with MedSAM-2 weights. All parameters were frozen except (i) the prompt encoder, (ii) the mask decoder, and (iii) the final ≈10% of layers in the image encoder. Let $f_{\theta }(x,b)$ denote the Endo-MedSAM output logits for slice x and prompt *b*. We optimized a combined binary cross-entropy (BCE) and Dice loss. Given logits $z=f_{\theta }(x,b)\;$ and probabilities $\hat{y}=\sigma (z)$, the element-wise BCE is

$$L_{\mathrm{BCE}}(z,y)=-\frac{1}{N}\overset{N}{\underset{i=1}{\sum }}\;[y_{i}\log({\hat{y}}_{i})+(1-y_{i})\log(1{\hat{y}}_{i})],$$

and the soft Dice coefficient is

$$\mathrm{Dice}(\hat{y},y)=\frac{2\sum_{i=1}^{N}\;{\hat{y}}_{i}y_{i}+\varepsilon }{\sum_{i=1}^{N}\;{\hat{y}}_{i}+\sum_{i=1}^{N}\;y_{i}+\varepsilon },$$

with $\varepsilon =10^{-6}$. The training loss was

$$L=L_{\mathrm{BCE}}+\lambda_{\mathrm{Dice}}(1-\mathrm{Dice}(\hat{y},y)),$$

with $\lambda_{\mathrm{Dice}}=0.5$. Optimization was performed using AdamW (learning rate $1\times 10^{-5}$, weight decay $4\times 10^{-5}$), batch size 4, and a maximum of 20 epochs. In each epoch we computed the mean slice-wise Dice on the validation set; the checkpoint with the highest validation Dice was retained.

For inference, the same preprocessing and normalization pipeline was applied. For a given test slice and uterus prompt (bounding box or point-based), the model produced a logit mask that was upsampled to 256×256 and thresholded at 0.5 to obtain a binary prediction. Slice-wise predictions were stacked to form a 3D mask for each subject. The 3D Dice coefficient between predicted volume *P* and ground-truth volume *G* was computed as

$$\mathrm{Dic}e_{3D}(P,G)=\frac{2|P\cap G|+\varepsilon }{|P|+|G|+\varepsilon }.$$

To complement the overlap-based Dice metric, we also computed the 95th percentile Hausdorff distance (HD95) at both the slice level and the subject-level 3D volume level. HD95 quantifies boundary agreement between the predicted and reference masks while reducing sensitivity to isolated outlier voxels; lower HD95 values indicate better structural consistency of the segmentation.

### 2.5 Evaluation setups and prompting strategies

To assess generalization across scanners, protocols, and centers, Endo-MedSAM was evaluated under three complementary training–testing setups, all defined at the patient level (no subject appears in more than one split). Prompt simulation (interactive setting): For all promptable experiments (Endo-MedSAM and the zero-shot MedSAM-2 baseline), bounding boxes and point prompts were programmatically generated from the reference uterus mask to simulate user-provided localization cues. Accordingly, results reflect interactive prompt-guided segmentation rather than fully automatic detection; in deployment, prompts would be provided by a clinician.

#### 2.5.1 Dataset splits

- **Setup 1 – Train on D2, test on D1:** The model was fine-tuned on the single-center dataset D2 and evaluated on the multi-center dataset D1 to test robustness to heterogeneous scanners and protocols.
- **Setup 2 – Train on D1, test on D2:** Training used only the multi-center D1 data and external validation was performed on the homogeneous clinical cohort D2.
- **Setup 3 – Joint training on D1** **+** **D2:** Both datasets were pooled and randomly partitioned at the patient level into a training set (≈75% of subjects), an internal validation set, and a held-out test set (≈25% of subjects), providing an estimate of performance when training on a combined, more diverse cohort.

#### 2.5.2 Prompting modes

For each dataset setup we trained three separate Endo-MedSAM models, differing only in the type of prompt supplied:

- **Bounding-box prompts:** A 2D bounding box was derived from the reference uterus mask and uniformly expanded in the x- and y-dimensions by a scale factor (s) sampled uniformly in [(1.2, 1.7)] during training to improve robustness to user variability. Validation and primary test experiments used a fixed scale (s = 1.5). The box was passed to the SAM-2 prompt encoder as a pair of corner points.
- **1-click point prompts:** A single positive point was sampled inside the uterus mask, near its centroid, and encoded as a foreground prompt. All background pixels were implicitly treated as non-uterus.
- **2-click point prompts:** One positive point was placed inside the uterus as above, and one negative point was sampled in the background region outside the mask but within the same slice. This combination encourages the model to both include the uterus and exclude adjacent pelvic structures.

Each prompting mode was trained and evaluated independently but used identical data splits, augmentation, and optimization settings, allowing a fair comparison of how different clinical interaction modes (box vs. single- or dual-click prompting) influence segmentation performance.

#### 2.5.3 Baseline models

As baselines, we compared Endo-MedSAM against (i) a prompt-guided foundation-model baseline and (ii) a strong fully automatic supervised baseline. First, we evaluated the original MedSAM-2 model in a zero-shot configuration using the official MedSAM-2 weights, without any fine-tuning on our datasets. To ensure a fair comparison, MedSAM-2 was evaluated in the same image-based, 2D slice-wise prompting setting used for Endo-MedSAM, and identical prompt definitions were applied (bounding box, 1-click, and 2-click; Section 2.5.2). Prompts were programmatically derived from the reference uterus mask to simulate user-provided localization cues (interactive segmentation). Second, we trained nnU-Net v2 in both 2D and 3D configurations (25, 26) as fully automatic baselines requiring no prompts. nnU-Net v2 is a self-configuring medical segmentation framework that automatically selects key preprocessing and training design choices (e.g., resampling, normalization, patch size, augmentation, and optimization) based on dataset properties. Both nnU-Net models were trained using the same patient-level splits and evaluated on the same held-out test sets as Endo-MedSAM, using the same slice-level and 3D Dice metrics.

#### 2.5.4 Bounding Box ablation study

To assess robustness to plausible variability in clinician-provided bounding boxes, we performed an ablation study on the bounding-box prompt definition while keeping the trained Endo-MedSAM weights fixed. Because all prompts in this retrospective study were programmatically derived from the reference uterus mask rather than collected from real users, this analysis was designed to approximate a range of realistic box tightness and centering errors under controlled conditions. The primary Endo-MedSAM model for bounding-box prompting was trained using tight reference-mask boxes with isotropic random expansion during training (s∼U(1.2,1.7)), and the main validation and test experiments used a fixed scale of s=1.5.

For the ablation, inference was repeated under four alternative prompt-generation conditions. First, we evaluated the previously used **fixed-scale isotropic box expansion** by varying the expansion factor applied to the tight reference box at test time (s∈{1.2,1.5,2.0}), enabling assessment of sensitivity to tighter or looser but still centered bounding boxes. In addition, we evaluated three **asymmetric perturbation modes** designed to relax the assumption that the uterus is perfectly centered within the box. In these asymmetric modes, the left, right, top, and bottom box boundaries were expanded non-uniformly. For a tight reference box with width *w* and height *h*, the perturbed box was defined as:

$$x_{1}^{{}^{\prime }}=x_{1}-\alpha_{L}w,x_{2}^{{}^{\prime }}=x_{2}+\alpha_{R}w,y_{1}^{{}^{\prime }}=y_{1}-\alpha_{T}h,y_{2}^{{}^{\prime }}=y_{2}+\alpha_{B}h,$$

where the final coordinates were clipped to the image boundaries.

Rather than sampling each side independently from a single uniform range, the asymmetric perturbations were generated using a mode-specific base expansion and an imbalance term along each axis, such that one side was expanded more than the opposite side in a controlled but randomized manner. This produced off-center, non-uniform boxes while avoiding unrealistically extreme perturbations.

We considered three asymmetric perturbation settings of increasing severity:

1. **Mild asymmetric expansion**, which introduced small directional imbalance and limited off-centering. For each axis, the base expansion fraction was sampled from approximately 0.11–0.15 and the imbalance magnitude from approximately 0.015–0.035, with resulting side-specific margins constrained to 0.07–0.22.
2. **Moderate asymmetric expansion**, which introduced greater asymmetry and moderate off-centering. For each axis, the base expansion fraction was sampled from approximately 0.15–0.20 and the imbalance magnitude from approximately 0.04–0.08, with resulting side-specific margins constrained to 0.08–0.30.
3. **Off-center asymmetric expansion**, which introduced the strongest directional bias and the largest center displacement while keeping the overall box size within a plausible range. For each axis, the base expansion fraction was sampled from approximately 0.16–0.21 and the imbalance magnitude from approximately 0.08–0.135, with resulting side-specific margins constrained to 0.06–0.34.

This ablation was performed across all three dataset setups using the same trained checkpoint in each setup, without any additional fine-tuning, so that observed differences reflected only prompt perturbation sensitivity. Quantitative results were summarized using the same slice-level Dice and subject-level 3D Dice metrics as in the primary experiments. For the asymmetric perturbation analysis. Overall, this analysis was intended to estimate how Endo-MedSAM responds to increasingly imperfect bounding-box localization cues, bridging the gap between idealized reference-derived prompts and more variable clinician-drawn prompts in practice.

## 3 Results

**Figure 2:**
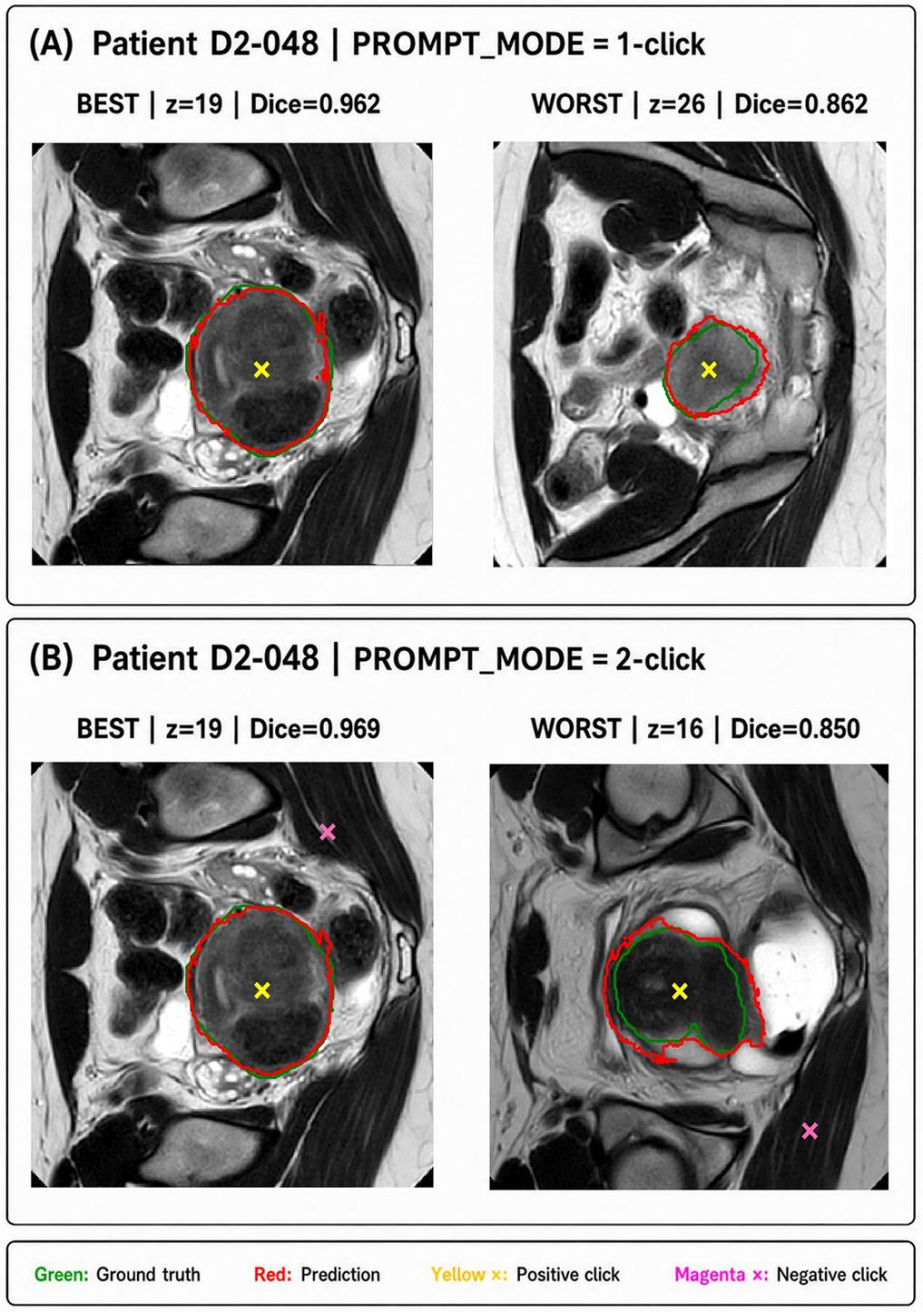
Single-patient qualitative example of uterus segmentation by Endo-MedSAM on pelvic MRI for Patient D2-048 from the D2 cohort. The figure is divided into two panels showing interactive click-prompting modes: **(A)** 1-click and **(B)** 2-click. Each panel presents the best-performing slice on the left and the worst-performing slice on the right, with the slice index (z) and Dice score displayed above each image. Green contours denote the ground-truth uterus mask, and red contours denote the Endo-MedSAM predicted segmentation. Colored cross markers indicate the interactive click prompts used for segmentation.

**Figure 3:**
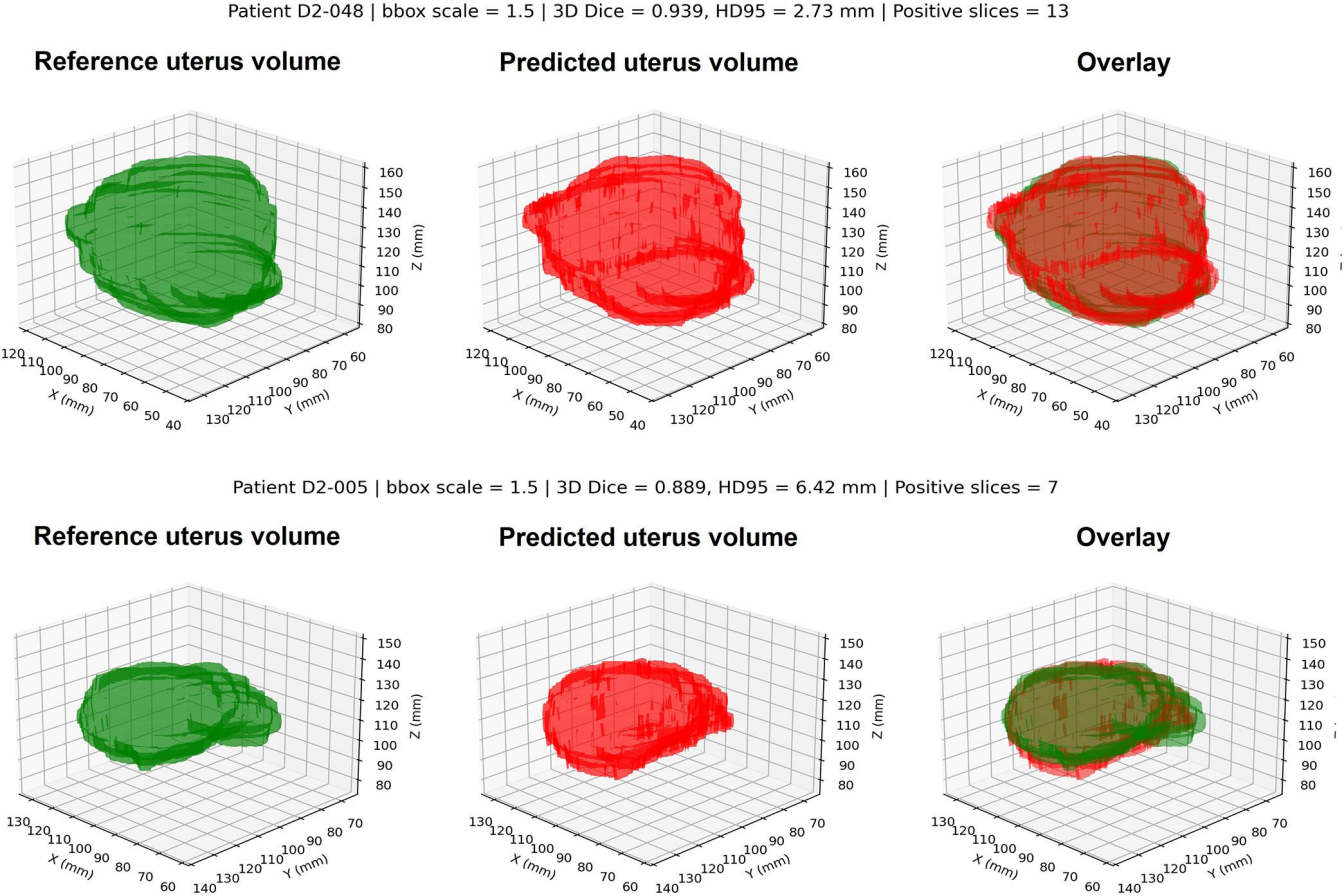
Example of 3D renderings of reconstructed uterus segmentations. Ground-truth and Endo-MedSAM predicted volumes are shown for representative test cases, to illustrate overall 3D shape agreement beyond slice-wise qualitative views and numerical Dice/HD95.

**Figure 4:**
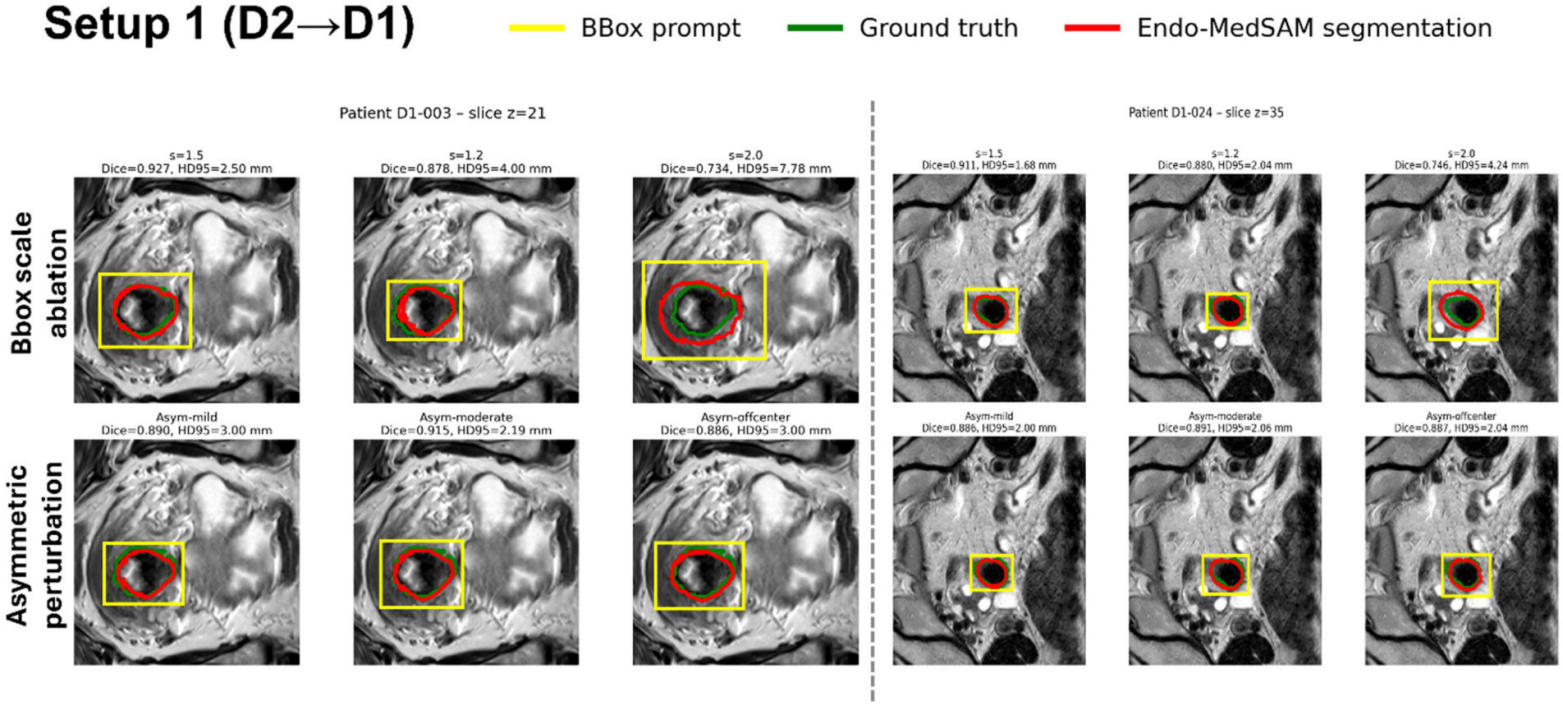
Ablation examples for setup 1 (D2→D1). Top row: bounding-box scale ablation using isotropic box expansion factors (s = 1.5), (1.2), and (2.0). Bottom row: asymmetric bounding-box perturbation ablation using Asym-mild, Asym-moderate, and Asym-offcenter prompts. Yellow boxes indicate the bounding-box prompt, green contours indicate the ground-truth uterus mask, and red contours indicate the Endo-MedSAM prediction.

**Figure 5:**
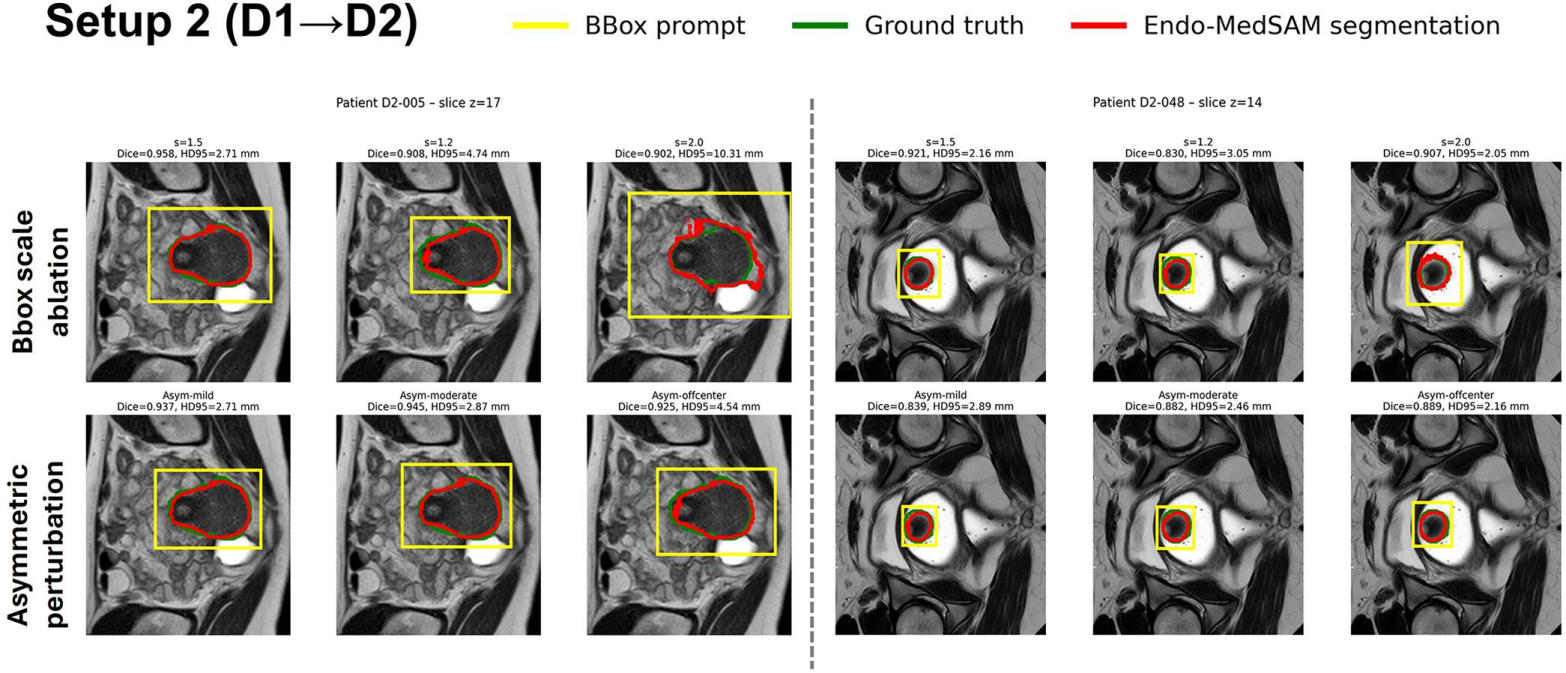
Ablation examples for setup 2 (D1→D2). Top row: bounding-box scale ablation using isotropic box expansion factors (s = 1.5), (1.2), and (2.0). Bottom row: asymmetric bounding-box perturbation ablation using Asym-mild, Asym-moderate, and Asym-offcenter prompts. Yellow boxes indicate the bounding-box prompt, green contours indicate the ground-truth uterus mask, and red contours indicate the Endo-MedSAM prediction.

This section reports uterus segmentation performance of Endo-MedSAM across three patient-level training–testing setups designed to assess cross-center generalization (Sections 3.1–3.3), followed by an analysis of prompting strategy effects (Section 3.4). Quantitative results are summarized in Table 2, and a representative qualitative example illustrating best- and worst-performing slices under interactive click prompting is shown in Figure 2. We additionally report an ablation study examining robustness to bounding-box perturbations across isotropic scale changes and asymmetric box perturbations (Section 3.5, Table 3, Figures 3–5).

**Table 2:** Uterus segmentation performance across evaluation setups and prompting strategies.

Experiment	Train → Test	Prompt	3D Dice (mean ± SD)	Slice Dice (mean ± SD)	3D HD95 (mm) (mean ± SD)	Slice HD95 (mm) (mean ± SD)
Endo-MedSAM (Setup 1)	D2 (single-center) → D1 (multicenter)	bbox	0.806 ± 0.102	0.795 ± 0.1485	8.693 ± 5.7599	10.875 ± 8.685
		1-click	0.681 ± 0.138	0.664 ± 0.239	13.730 ± 6.750	16.012 ± 11.494
		2-click	0.691 ± 0.124	0.671 ± 0.223	13.705 ± 6.393	16.233 ± 10.888
Endo-MedSAM (Setup 2)	D1 (multicenter) → D2 (single-center)	bbox	0.861 ± 0.055	0.856 ± 0.088	5.295 ± 2.339	6.586 ± 4.163
		1-click	0.737 ± 0.155	0.716 ± 0.210	10.935 ± 6.834	13.247 ± 9.668
		2-click	0.746 ± 0.135	0.724 ± 0.198	10.792 ± 6.509	12.966 ± 9.194
Endo-MedSAM (Setup 3)	D1 + D2 (75% train) → D1 + D2 (25% test)	bbox	**0.879 ± 0.060**	**0.863 ± 0.095**	**4.810 ± 2.806**	8.025 ± 5.845
		1-click	0.758 ± 0.138	0.751 ± 0.199	11.106 ± 6.638	13.512 ± 9.215
		2-click	0.758 ± 0.124	0.758 ± 0.189	11.576 ± 5.861	13.542 ± 9.115
Zero-shot MedSAM-2	Pooled test sets	bbox	0.541 ± 0.147	0.538 ± 0.211	17.780 ± 6.310	20.902 ± 12.327
		1-click	0.490 ± 0.192	0.442 ± 0.294	17.319 ± 9.732	21.474 ± 13.938
		2-click	0.481 ± 0.185	0.433 ± 0.278	17.452 ± 9.848	21.381 ± 13.534
nnU-Net v2 (3D)	D1 + D2 (75% train) → D1 + D2 (25% test)	—	0.587 ± 0.289	0.4776 ± 0.2540	21.145 ± 17.485	19.3136 ± 11.6165
nnU-Net v2 (2D)	D1 + D2 (75% train) → D1 + D2 (25% test)	—	0.539 ± 0.262	0.433 ± 0.227	23.865 ± 15.639	24.050 ± 15.205

Mean ± standard deviation are reported for subject-level 3D Dice, slice-level Dice, subject-level 3D HD95, and slice-level HD95 for Endo-MedSAM under three train-test setups using bounding-box, 1-click, and 2-click prompts, alongside zero-shot MedSAM-2 and nnU-Net v2 (2D/3D) baselines. Setup 1 evaluates cross-center generalization from single-center training (D2) to multicenter testing (D1), Setup 2 evaluates the reverse direction, and Setup 3 evaluates mixed-center training and testing. Higher Dice and lower HD95 indicate better segmentation performance.

Bold values indicate the best results.

**Table 3:** Bounding-box prompt ablation results across the three experimental setups.

Setup	Ablation type	Condition	3D Dice (mean ± SD)	Slice Dice (mean ± SD)	3D HD95 (mm) (mean ± SD)	Slice HD95 (mm) (mean ± SD)
Setup 1 (D2→D1)	bbox scale	s = 1.2	0.743 ± 0.062	0.731 ± 0.117	8.860 ± 4.450	11.121 ± 7.236
		s = 1.5	0.806 ± 0.102	0.795 ± 0.1485	8.693 ± 5.7599	10.875 ± 8.685
		s = 2.0	0.711 ± 0.113	0.693 ± 0.157	12.296 ± 5.817	15.746 ± 10.026
	asymmetric perturbation	Asym-mild	0.764 ± 0.072	0.752 ± 0.128	8.670 ± 4.872	11.016 ± 7.547
		Asym-moderate	0.785 ± 0.085	0.775 ± 0.137	8.747 ± 5.457	11.137 ± 8.095
		Asym-offcenter	0.766 ± 0.086	0.756 ± 0.140	9.015 ± 5.536	12.092 ± 8.467
Setup 2 (D1→D2)	bbox scale	s = 1.2	0.792 ± 0.061	0.789 ± 0.090	6.562 ± 2.147	8.056 ± 4.390
		s = 1.5	0.861 ± 0.055	0.856 ± 0.088	5.295 ± 2.339	6.586 4.163
		s = 2.0	0.805 ± 0.067	0.803 ± 0.112	9.237 ± 4.639	10.592 ± 6.747
	asymmetric perturbation	Asym-mild	0.812 ± 0.061	0.806 ± 0.094	6.218 ± 2.164	7.690 ± 4.430
		Asym-moderate	0.832 ± 0.059	0.827 ± 0.096	5.910 ± 2.139	7.367 ± 4.255
		Asym-offcenter	0.825 ± 0.057	0.820 ± 0.092	6.308 ± 2.312	7.887 ± 4.465
Setup 3 (pooled D1 & D2)	bbox scale	s = 1.2	0.824 ± 0.060	0.817 ± 0.112	5.990 ± 2.419	8.537 ± 5.418
		s = 1.5	0.879 ± 0.060	0.863 ± 0.095	4.810 ± 2.806	8.025 ± 5.845
		s = 2.0	0.804 ± 0.059	0.801 ± 0.103	9.329 ± 4.707	11.660 ± 7.280
	asymmetric perturbation	Asym-mild	0.844 ± 0.058	0.833 ± 0.111	5.574 ± 2.417	8.306 ± 5.537
		Asym-moderate	0.857 ± 0.061	0.844 ± 0.112	5.316 ± 2.535	8.332 ± 5.635
		Asym-offcenter	0.838 ± 0.063	0.827 ± 0.113	5.925 ± 2.570	8.999 ± 5.721

### 3.1 Setup 1: training on single-center D2, testing on multicenter D1

When Endo-MedSAM was trained on the single-center D2 cohort and evaluated on the multicenter D1 cohort, the best performance was obtained with bounding-box prompting, achieving a mean 3D Dice of 0.806 ± 0.102 and a mean slice Dice of 0.795 ± 0.149. The corresponding mean 3D and slice HD95 values were 8.693 ± 5.760 mm and 10.875 ± 8.685 mm, respectively. Performance was lower with interactive point prompting. Under the 1-click setting, mean 3D Dice was 0.681 ± 0.138 and mean slice Dice was 0.664 ± 0.239, while the 2-click setting yielded mean 3D and slice Dice values of 0.691 ± 0.124 and 0.671 ± 0.223, respectively. HD95 values were also higher for both click-based modes than for bounding-box prompting (Table 2).

### 3.2 Setup 2: training on multicenter D1, testing on single-center D2

When the model was trained on the multicenter D1 cohort and externally evaluated on the single-center D2 cohort, Endo-MedSAM again achieved its highest performance with bounding-box prompting. In this setting, mean 3D Dice reached 0.861 ± 0.055 and mean slice Dice reached 0.856 ± 0.088, with corresponding mean 3D and slice HD95 values of 5.295 ± 2.339 mm and 6.586 ± 4.163 mm, respectively. Performance with point-based prompting remained lower but improved relative to Setup 1. For 1-click prompting, mean 3D Dice was 0.737 ± 0.155 and mean slice Dice was 0.716 ± 0.210, whereas 2-click prompting yielded mean 3D Dice of 0.746 ± 0.135 and mean slice Dice of 0.724 ± 0.198. Both point-based settings showed higher HD95 values than the bounding-box condition (Table 2).

### 3.3 Setup 3: mixed-cohort training and testing

The highest overall performance was observed in the mixed-cohort setting, where D1 and D2 were pooled and split at the patient level into training and held-out test subsets. In this configuration, bounding-box prompting produced a mean 3D Dice of 0.879 ± 0.060 and a mean slice Dice of 0.863 ± 0.095, with mean 3D and slice HD95 values of 4.810 ± 2.806 mm and 8.025 ± 5.845 mm, respectively. Point-based prompting again resulted in lower overlap performance. Under the 1-click setting, mean 3D Dice was 0.758 ± 0.138 and mean slice Dice was 0.751 ± 0.199, while the 2-click setting produced mean 3D Dice of 0.758 ± 0.124 and mean slice Dice of 0.758 ± 0.189. Across all prompting modes, the mixed training and testing configuration yielded the strongest quantitative results among the three setups (Table 2).

### 3.4 Effect of prompting strategy

Across all three experimental setups, bounding-box prompting consistently yielded the highest segmentation accuracy and the lowest boundary-distance error. Mean 3D Dice for bounding-box prompting ranged from 0.806 to 0.879, compared with 0.681 to 0.758 for 1-click prompting and 0.691 to 0.758 for 2-click prompting. A similar trend was observed for slice-level Dice, which ranged from 0.795 to 0.863 for bounding-box prompting, 0.664 to 0.751 for 1-click prompting, and 0.671 to 0.758 for 2-click prompting. The corresponding HD95 values were also consistently lower for bounding-box prompting than for either point-based mode. Differences between 1-click and 2-click prompting were modest across setups, with 2-click prompting showing slightly higher Dice than 1-click prompting in Setups 1 and 2, while both prompting modes produced nearly identical 3D Dice in Setup 3. Representative qualitative examples of best- and worst-performing cases under click-based prompting are shown in Figure 2. To further aid interpretation of volumetric performance, Figure 3 presents representative 3D renderings of reconstructed predicted and reference uterus segmentations. These visualizations help illustrate how the reported Dice and HD95 values correspond to overall volumetric shape agreement and localized contour differences in the reconstructed uterus. Together, they complement the slice-wise qualitative results in Figure 2 and provide a more intuitive assessment of anatomical segmentation quality.

Compared with zero-shot MedSAM-2, Endo-MedSAM showed higher performance across all prompting strategies. On the pooled test sets, zero-shot MedSAM-2 achieved mean 3D Dice values of 0.541 with bounding-box prompting, 0.490 with 1-click prompting, and 0.481 with 2-click prompting, all below the corresponding Endo-MedSAM results. In the mixed-cohort setting, Endo-MedSAM with bounding-box prompting also outperformed the fully automatic nnU-Net baselines, which achieved mean 3D Dice values of 0.587 for nnU-Net v2 (3D) and 0.539 for nnU-Net v2 (2D) (Table 2).

### 3.5 Ablation analysis: robustness to bounding-box perturbations

Table 3 summarizes the bounding-box ablation results across all three setups. For isotropic scale perturbations, the highest performance was consistently observed at the validation-matched setting of s = 1.5. In Setup 1, mean 3D Dice increased from 0.743 ± 0.062 at s = 1.2 to 0.806 ± 0.102 at s = 1.5, then decreased to 0.711 ± 0.113 at s = 2.0. In Setup 2, the corresponding values were 0.792 ± 0.061, 0.861 ± 0.055, and 0.805 ± 0.067. In Setup 3, mean 3D Dice was 0.824 ± 0.060 at s = 1.2, 0.878 ± 0.060 at s = 1.5, and 0.804 ± 0.059 at s = 2.0. A similar pattern was observed for slice Dice and HD95, with s = 1.5 generally yielding the lowest boundary-distance errors.

**Figure 6:**
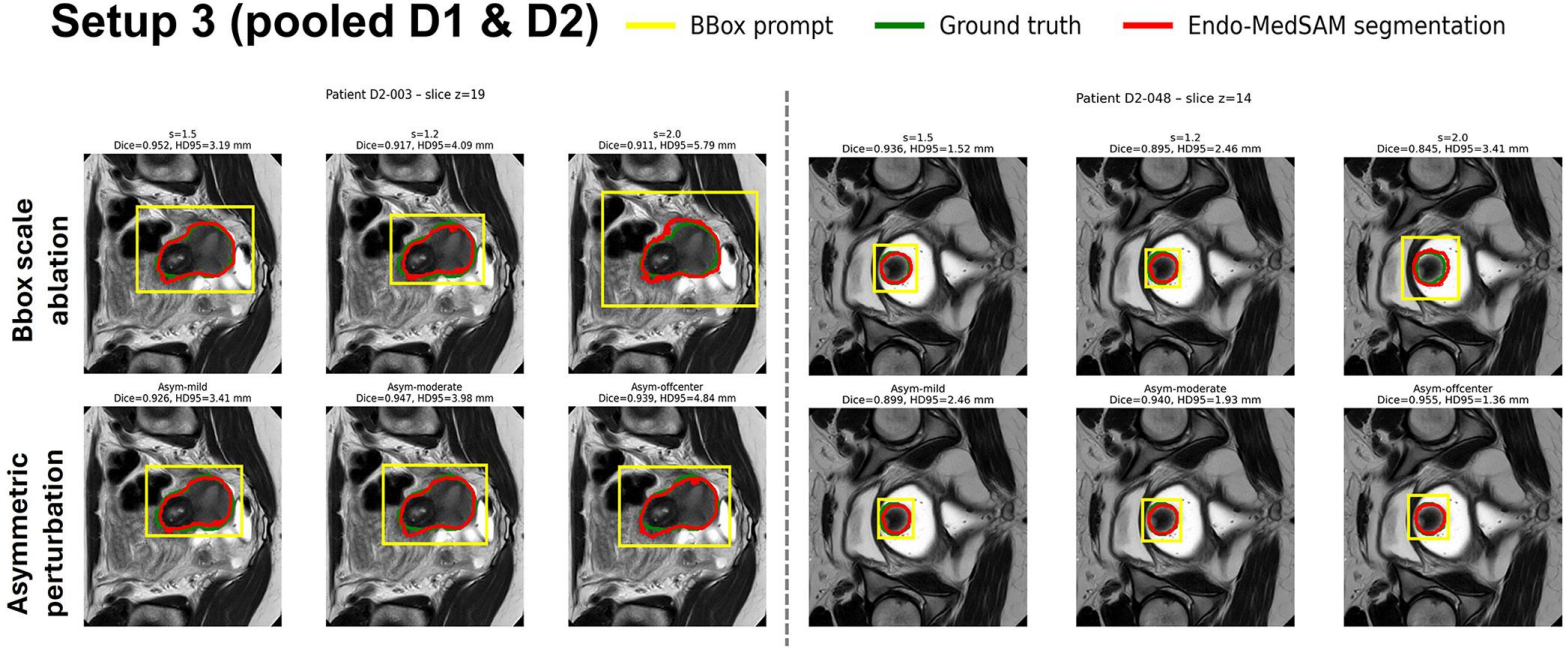
Ablation examples for setup 3 (pooled D1 & D2). Top row: bounding-box scale ablation using isotropic box expansion factors (s = 1.5), (1.2), and (2.0). Bottom row: asymmetric bounding-box perturbation ablation using Asym-mild, Asym-moderate, and Asym-offcenter prompts. Yellow boxes indicate the bounding-box prompt, green contours indicate the ground-truth uterus mask, and red contours indicate the Endo-MedSAM prediction.

Under asymmetric perturbations, performance remained below the s = 1.5 centered-box condition but above or near the tighter isotropic setting in most cases. In Setup 1, mean 3D Dice ranged from 0.764 to 0.785 across the three asymmetric modes. In Setup 2, the corresponding range was 0.812 to 0.832, and in Setup 3 it was 0.838 to 0.857. Among the asymmetric settings, Asym-moderate produced the highest 3D Dice in all three setups. Representative qualitative examples of isotropic and asymmetric box perturbations are shown in Figures 4–6.

## 4 Discussion

### 4.1 Principal findings

This study demonstrates that a uterus-focused adaptation of a promptable medical vision foundation model can achieve robust segmentation performance on pelvic MRI in women with endometriosis, despite the anatomical complexity of this population. Across all three evaluation setups, Endo-MedSAM consistently performed best with bounding-box prompting, achieving mean 3D Dice values of 0.806 in Setup 1, 0.861 in Setup 2, and 0.879 in Setup 3, with corresponding slice Dice values of 0.795, 0.856, and 0.863. These results indicate that the model can generalize across both heterogeneous multicenter data and homogeneous single-center data, while benefiting further from training on a pooled cohort spanning both domains. The strongest performance in Setup 3 is expected, as mixed training exposes the model to a broader range of scanner characteristics, acquisition protocols, uterine positions, and disease-related distortions, thereby improving representation robustness and reducing domain-specific bias.

A second important finding is the strong and consistent effect of prompting strategy. Bounding-box prompting substantially outperformed both 1-click and 2-click prompting in every setup, not only in overlap metrics but also in boundary accuracy as reflected by lower HD95 values. This suggests that, for uterus segmentation on endometriosis MRI, a coarse spatial extent cue is more informative than sparse point guidance alone. The uterus often occupies a relatively large but variably shaped pelvic structure, and in endometriosis its boundaries may be distorted by adhesions, fibroids, adenomyosis, mass effect, or altered uterine position. Under these conditions, a box prompt likely provides the model with a more stable contextual prior regarding target extent and surrounding anatomy, whereas one or two points may be insufficient to disambiguate the uterus from adjacent soft-tissue structures with similar signal characteristics. The relatively modest difference between 1-click and 2-click prompting further supports the interpretation that adding a single background click improves local exclusion only slightly when the overall anatomic search space remains underconstrained.

The comparison with baselines also strengthens the main conclusion of the study. Endo-MedSAM consistently outperformed zero-shot MedSAM-2 across all prompting modes, indicating that uterus-specific adaptation is necessary for this task and that generic medical promptable representations alone are insufficient in the presence of endometriosis-related pelvic distortion. In the pooled setting, Endo-MedSAM with bounding-box prompting also outperformed both nnU-Net v2 baselines, suggesting that promptable fine-tuning of a foundation model can provide an advantage over strong conventional supervised segmentation pipelines in this application. Taken together, these findings support the central premise of the work: adapting MedSAM-2 to the target anatomy and clinical domain yields a practically useful interactive segmentation model for challenging endometriosis MRI.

### 4.2 Robustness, generalization, and interpretation of the ablation findings

An important contribution of this study is the explicit evaluation of robustness to prompt variability. Because the main bounding-box experiments used reference-derived prompts, the ablation study was essential to test whether performance remained stable under more realistic deviations in prompt tightness and centering. Two main patterns emerged. First, across all three setups, the best performance was consistently observed at s = 1.5, which matched the fixed validation setting and lay within the range of random box expansion used during training. Performance declined at s = 1.2 and more noticeably at s = 2.0, indicating that the model is tolerant to moderate prompt variation but remains sensitive to shifts away from the training-aligned prompt distribution. This is an expected behavior for prompt-conditioned segmentation models: while they should not require pixel-perfect prompting, their highest performance is likely to occur when inference prompts resemble those encountered during optimization.

Second, the asymmetric perturbation analysis provides a more clinically meaningful perspective on prompt robustness than isotropic scaling alone. Even when the uterus was no longer perfectly centered within the prompt and the box margins became directionally imbalanced, Endo-MedSAM maintained reasonably strong performance across all setups. Although asymmetric conditions generally remained below the centered s = 1.5 condition, they often performed comparably to, or better than, tighter isotropic boxes. This suggests that the model does not simply rely on idealized, perfectly centered prompts, but can tolerate a plausible degree of off-centering and non-uniform box drawing. From a clinical standpoint, this is important because real user-drawn boxes are unlikely to be symmetric or perfectly aligned. The ablation results therefore narrow the gap between controlled prompt simulation and realistic interactive use, while still showing that excessively loose prompts can degrade segmentation quality, especially under stronger domain shift.

The differences observed across Setups 1, 2, and 3 also help interpret the model's generalization behavior. Setup 1, in which the model was trained on the more homogeneous D2 cohort and tested on the more heterogeneous D1 cohort, was the most challenging configuration and showed the lowest performance overall. This likely reflects the difficulty of transferring from a narrow training distribution to a broader multicenter test domain containing greater variability in scanners, protocols, and anatomy. In contrast, Setup 2 showed stronger external generalization, suggesting that training on the more diverse D1 cohort learned features that transferred better to the single-center D2 test set. Setup 3 then produced the highest results, reinforcing the value of exposure to both domains during training. This pattern is clinically relevant because it suggests that, for deployment across institutions, diverse multi-domain training data may be more important than maximizing performance within a single acquisition setting.

Overall, these findings indicate that Endo-MedSAM is not only accurate under idealized prompting, but also reasonably robust to realistic perturbations in box definition.

### 4.3 Clinical implications and limitations

Endo-MedSAM enables fast, reproducible uterus segmentation that can reduce manual contouring burden and help standardize volumetry across sites, supporting downstream quantitative MRI analyses and preoperative planning workflows in endometriosis care. At the same time, the results clarify that prompt design remains an important determinant of performance. In practice, this means that Endo-MedSAM is best viewed as a prompt-guided interactive segmentation tool rather than a prompt-invariant automatic segmenter. Its clinical usefulness would therefore depend on pairing the model with simple, consistent user interaction, particularly bounding-box inputs that approximate the scale and framing encountered during development.

Several limitations merit consideration. First, prompts were simulated from reference masks, representing an interactive setting rather than fully automatic detection. Second, adaptation was performed slice-wise using an image-based formulation, and MedSAM-2's video-style propagation capabilities were not exploited. Third, the cohort size was modest (74 subjects) and restricted to T2-weighted sequences, which may limit generalizability across broader imaging protocols and disease phenotypes. A further limitation is that the MRI data were acquired as native axial volumes with anisotropic voxel spacing, so sagittal and coronal views would require resampling rather than providing true additional plane-specific information. Accordingly, we restricted the present analysis to the axial plane.

### 4.4 Future work

Several extensions could improve clinical utility and robustness. Incorporating multi-sequence MRI inputs (e.g., T2 fat-suppressed, T1, and T1 fat-suppressed) may provide complementary tissue contrast to better handle coexisting fibroids/adenomyosis and variable uterine boundaries. Expanding from uterus-only segmentation to multi-organ pelvic segmentation (e.g., ovaries, endometriomas, bladder, rectum, and other relevant structures) would enable richer anatomical context and support endometriosis phenotyping and surgical planning. In addition, future studies should evaluate multi-view or fully 3D fusion approaches on isotropic or dedicated multi-planar MRI datasets, where cross-plane information may be more reliable and potentially improve volumetric segmentation accuracy. Finally, evaluation on larger and more diverse datasets that include additional sites, vendors, and patient subgroups will be essential to quantify robustness under real world domain shifts and support reliable deployment.

## 5 Conclusion

Endo-MedSAM adapts the MedSAM-2 vision foundation model for uterus segmentation on pelvic MRI in women with endometriosis and demonstrates robust performance across both heterogeneous multicenter and homogeneous single-center cohorts. By fine-tuning the prompt encoder, mask decoder, and a small tail of the image encoder, Endo-MedSAM substantially improves over zero-shot MedSAM-2, achieving high volumetric accuracy with bounding-box prompts and maintaining competitive performance with low-interaction point prompts. These findings support practical interactive segmentation workflows that can reduce manual contouring time, improve reproducibility across scanners and sites, and enable standardized volumetry and quantitative MRI analyses to support endometriosis care. Future work will evaluate multi-sequence MRI inputs, expand to multi-organ pelvic segmentation, and validate performance in larger, more diverse endometriosis datasets.

## Code availability

The code for the Endo-MedSAM model is publicly available at https://github.com/rawan-alsaad/EndoMedSAM.

## Data Availability

The data used in this study are publicly available and can be accessed through the Zenodo repository at https://zenodo.org/records/13749613.
