# Supplementary material for: Endo-MedSAM: a promptable vision foundation model adaptation for uterus segmentation on pelvic MRI in endometriosis
Source: Front Reprod Health. 2026 May 25;8:1790980. doi: 10.3389/frph.2026.1790980 (PMC13244484; doi:10.3389/frph.2026.1790980)
Supplement: Supplementary file 1 [file supplementaryfile1.pdf]

Supplementary Material 1: Additional Qualitative Examples of Model Failure

To further characterize model behavior beyond the main qualitative figure, we included additional appendix examples showing representative failure cases. These examples suggest that segmentation errors were mainly associated with three recurring scenarios: (1) very small uterine cross-sections on edge slices, (2) weak or ambiguous tissue boundaries, and (3) anatomically distorted or displaced uterus morphology with close proximity to adjacent pelvic structures. Thus, failures were not explained by one factor alone, but rather by the combination of limited visible target extent and challenging local anatomy.

In the first failure example (Figure S.1a), the uterus is represented by only a very small cross-sectional area, likely corresponding to a peripheral slice near the superior or inferior extent of the organ. In this setting, the target occupies very few pixels, making overlap-based metrics highly sensitive to even minor contour offsets. This explains why the Dice score is low despite a very small HD95, indicating that the prediction remained spatially close to the reference mask but did not sufficiently overlap it.

In the second failure example (Figure S.1b), the model prediction extends along an elongated adjacent structure rather than matching the more compact reference contour. This pattern suggests confusion caused by distorted uterine anatomy, off-center morphology within the prompted region, and locally ambiguous boundaries with neighboring pelvic tissues. Such errors are qualitatively consistent with the anatomical complexity described in endometriosis, where adhesions, displacement, fibrotic change, adenomyosis, or coexisting lesions may alter the expected appearance and location of the uterus on individual slices.

Overall, these appendix examples indicate that model failures were most commonly related to partial-volume effects on small slices and boundary ambiguity under distorted pelvic anatomy, rather than simple random segmentation errors.

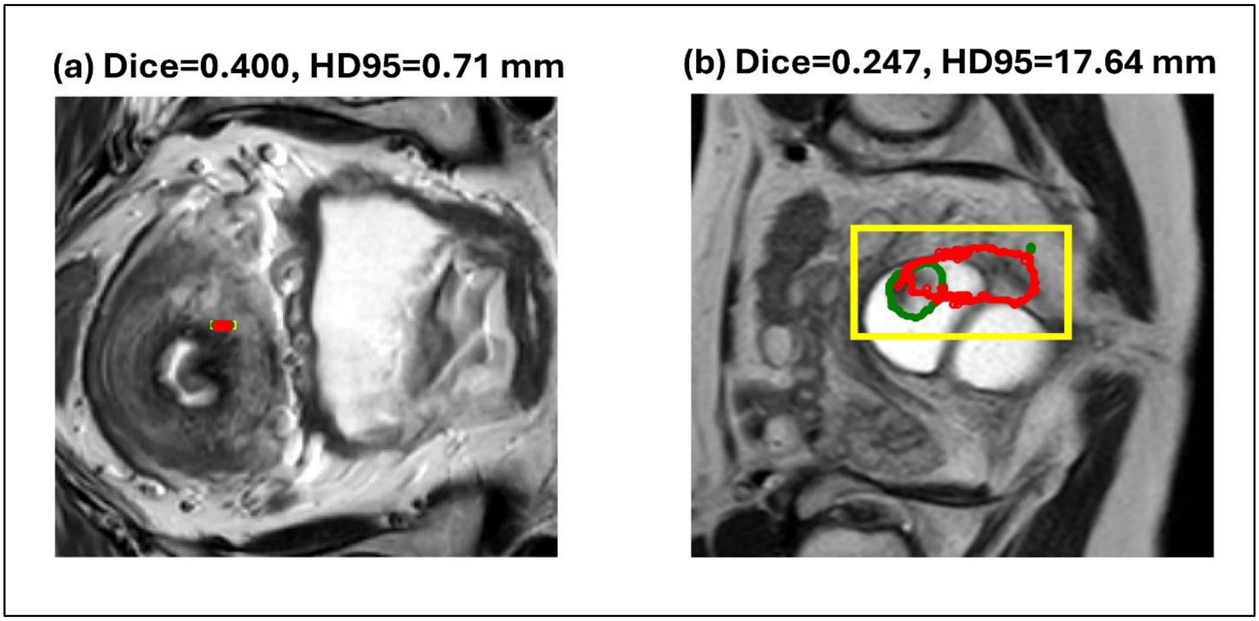

Figure S1. Additional qualitative failure examples for Endo-MedSAM.
